# Supplementary material for: Digital Trends in the Italian Beer Market: A Time-Series and Search Engine Optimisation Analysis of Gluten-Free and Low/No-Alcohol Beers
Source: Foods. 2025 Nov 5;14(21):3789. doi: 10.3390/foods14213789 (PMC12608848; doi:10.3390/foods14213789)
Supplement: Supplementary file 1 [file foods-14-03789-s001.zip › foods-3942264-supplementary.pdf]

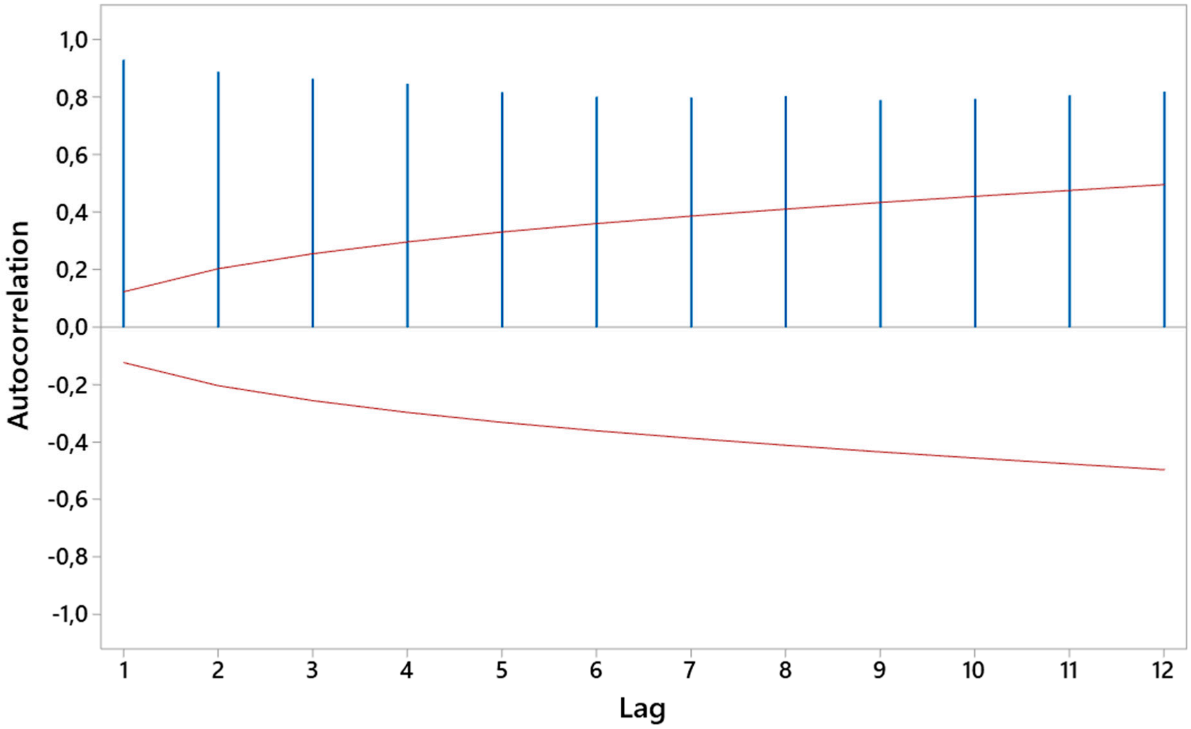

Figure S1. Autocorrelation function of the GFB keyword at a 12-month lag. Author's elaboration based on GT data

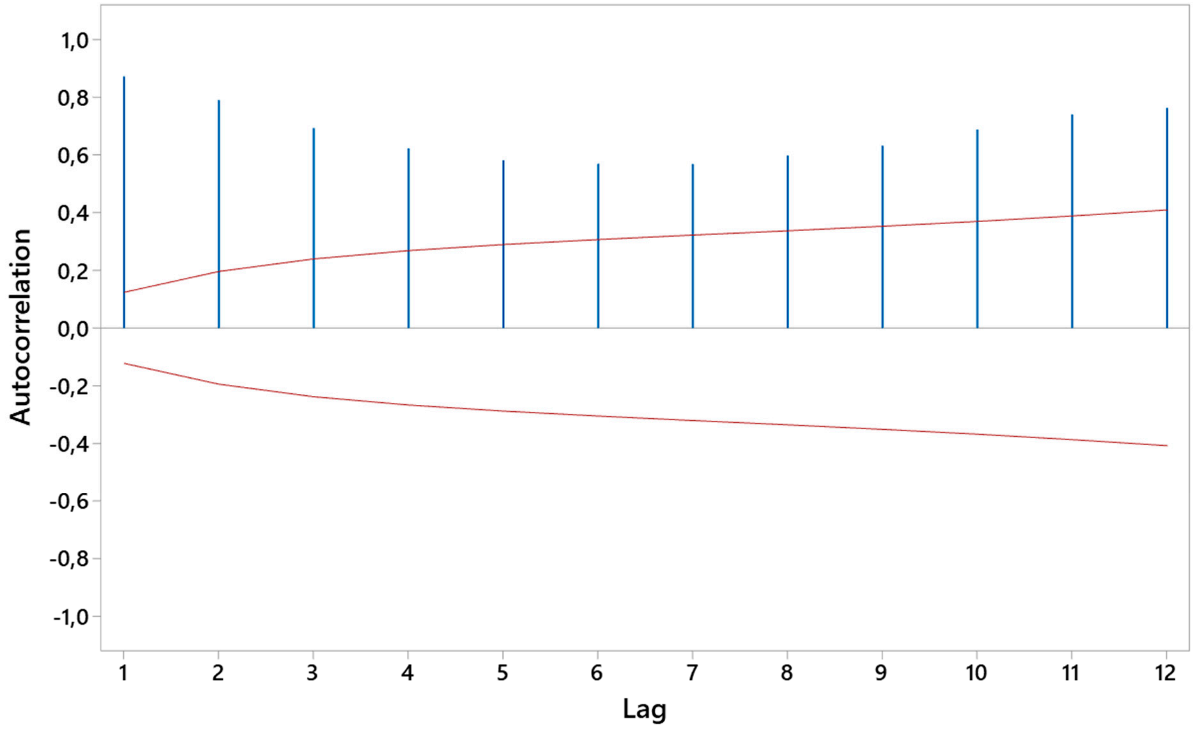

Figure S2. Autocorrelation function of the NABLAB keyword at a 12-month lag. Author's elaboration based on GT data
